# Factors associated with Finnish home care workers job satisfaction

**DOI:** 10.1093/eurpub/ckac131.375

**Published:** 2022-10-25

**Authors:** S Ruotsalainen, V Väisänen, L Corneliusson, A Noro, T Sinervo

**Affiliations:** Welfare State Research and Reform, Finnish Institute for Health and Welfare, Helsinki, Finland

## Abstract

**Background:**

In Finland, home care is seen as a primary form of care for older people. However, rising numbers of clients with increasingly complex conditions have led to deteriorated working conditions and poor job satisfaction among home care staff. In this study, we examined if greater amount of direct care time, higher team autonomy, number of unique clients, and client’s need for care are associated with job satisfaction.

**Methods:**

Data for the study was collected in October 2021. The amount of direct care and number of unique clients were obtained from a staff time measurement in home care units. The level of team autonomy was obtained from a survey sent to managers of participating organizations. Demographic information and a score for job satisfaction were retrieved from a survey for workers in the participating organizations. Client’s need for care was based on Resident Assessment Instrument (RAI). Data was analyzed using multiple linear regression.

**Results:**

The number of respondents was 387, of which the majority were practical nurses 307 (79%). Higher team autonomy was associated with better job satisfaction, whereas higher number of unique clients and higher amount of direct care time were associated with poorer job satisfaction. The models were adjusted with clients’ care needs, but these effects will be further examined.

**Conclusions:**

The results demonstrated that the workers are more satisfied if there is enough time to perform the work, they have adequate number of clients, and teams have autonomy over their work. This seems to be the case despite the level of clients’ need for care, however this needs further analysis. By increasing job satisfaction, better retention of care workers and attraction of care work might be achieved.

**Key messages:**

• Higher team autonomy is associated with care worker’s job satisfaction.

• Enhancing care continuity is important, therefore, when planning home care workers’ workday, it is important to consider that the workers could work with clients they know.

